# Maternal factors for neural tube defects in offspring: An umbrella review

**DOI:** 10.1515/med-2024-1061

**Published:** 2024-10-14

**Authors:** Hoda Arabzadeh, Ensiyeh Jenabi, Seyedeh Zahra Masoumi

**Affiliations:** Student Research Committee, Hamadan University of Medical Sciences, Hamadan, Iran; Mother and Child Care Research Center, Hamadan University of Medical Sciences, Hamadan, Iran; Autism Spectrum Disorders Research Center, Hamadan University of Medical Sciences, Hamadan, Iran; Department of Midwifery, School of Nursing and Midwifery, Mother and Child Care Research Center, Hamadan University of Medical Sciences, Hamadan, Iran

**Keywords:** neural tube defects, umbrella review, risk factor, maternal, pregnancy

## Abstract

**Objective:**

We conducted an umbrella review focusing on maternal risk factors during pregnancy associated with neural tube defects (NTDs).

**Methods:**

Our search was in databases PubMed, Scopus, and Web of Science. We specifically targeted meta-analyses examining maternal factors during pregnancy in relation to NTDs. The comparison involved assessing metrics such as odds ratio (OR) or related risk ratios reported in the included studies, as well as parameters like heterogeneity (*I*²), 95% prediction interval, small-study effects, excess significance biases, and sensitivity analysis.

**Results:**

Three risk factors for fetal NTDs, namely hyperthermia with an OR of 1.92, obesity with an OR of 1.68, and passive smoking with an OR of 1.90, were classified as highly suggestive evidence (Class II). Influenza, with an OR of 3.33, was considered a risk factor with suggestive evidence (Class III). Multivitamin supplementation during pregnancy, with an OR of 0.76, and low maternal vitamin B12, with an OR of 2.41, were categorized as weak evidence (Class IV).

**Conclusion:**

We identified four risk factors including hyperthermia, influenza, obesity, and passive smoking as suggestive or highly suggestive evidence for NTDs. Low maternal vitamin B12 was identified as a risk factor for NTDs, supported by weak evidence.

## 1 Introduction

Neural tube defects (NTDs) result from the incomplete closure of the neural tube between the 21st and 28th days after conception, making them one of the most common congenital malformations [1]. Globally, over 300,000 neonates are born with NTDs each year, leading to approximately 88,000 annual fatalities [2]. NTDs can result in miscarriages, infant mortality, and significant lifelong disabilities for affected children [3]. NTDs can be detected through straightforward prenatal testing methods, such as ultrasound imaging or screening for maternal serum alpha-fetoprotein (AFP) levels [4].

The causes of NTDs are multifaceted, involving both genetic and environmental risk factors. A family history of malformation was reported in cases with NTDs. The environmental risk factors for NTDs are consanguinity, consumption of fenugreek or other plants, diabetes, medication, maternal passive smoking, maternal obesity during pregnancy, maternal hyperthermia, low maternal vitamin B12 levels, maternal influenza, and the absence of multivitamin supplementation during pregnancy [5–11].

Tests to help diagnose NTDs before birth: blood test *t* measures the amount of AFP, fetal (prenatal) ultrasound recommends ultrasounds during the first trimester (11–14 weeks of pregnancy) and second trimester (18–22 weeks of pregnancy) and amniocentesis [12]. While treatment is often available, these screening methods may have disadvantages. It may be due to the unavailability or inadequacy of diagnostic tests that cause experimental misdiagnoses. The need of the hour is early diagnosis at the point of care of NTD patients [13].

Although various meta-analyses have been conducted for many maternal-related risk factors, an umbrella review study regarding maternal risk factors associated with NTDs in offspring has not been carried out to date. By conducting an umbrella review, researchers can obtain a broader understanding of the existing evidence, identify agreements or discrepancies among different systematic reviews, and assess the overall power and quality of the evidence base in a specific subject. Therefore, this study aims to comprehensively examine the meta-analyses of maternal risk factors during pregnancy associated with NTDs in offspring.

## 2 Materials and methods

On November 3, 2023, we officially registered the study protocol for the ongoing umbrella systematic review with the International Prospective Register of Systematic Reviews. The review strictly adhered to the Preferred Reporting Items for Systematic Reviews and Meta-Analyses guidelines [14]. The registration number for the study protocol is CRD42023475324.

### 2.1 Inclusion and exclusion criteria

In this umbrella review, we incorporated systematic reviews employing cohort or case–control designs. We deliberately excluded conference abstracts, letter to the editors, and original articles from our selection criteria. Additionally, genetic factors and maternal marker linked to fetal NTDs and animal studies were intentionally omitted from our consideration. Our primary focus centered on meta-analyses investigating maternal factors during pregnancy associated with fetal NTDs. Each meta-analysis earned inclusion in our study if it provided essential data for cumulative analysis; studies failing to meet this criterion were excluded. In cases where two or more meta-analyses pertained to a specific factor, our selection prioritized the study that encompassed the largest number of original studies.

### 2.2 Search strategy

To identify relevant systematic reviews on maternal factors during pregnancy associated with fetal NTDs, we implemented a search strategy that involved combining search terms using logical operators (i.e., AND/OR). Our search was conducted across three prominent international databases: PubMed, Scopus, and Web of Science. We systematically searched for articles published from the inception of these databases until October 23, 2023, without imposing any language or time restrictions. Detailed information regarding the search strategy is available in the supplementary material, specifically in Table S1.

### 2.3 Selection of studies

In the study selection process, two independent authors, E.J. and H.A., undertook the screening of titles and abstracts, as well as full texts, to assess their suitability for inclusion. Any discrepancies that emerged during this selection process were resolved through negotiation and discussion.

To ensure a thorough review, the authors extended their search beyond the initial database results by examining the reference lists of potentially relevant studies, aiming to identify any additional studies that might have been overlooked.

The study selection process employed a PECO model (population, exposure, comparison, and outcome). The population of interest comprised systematic reviews focusing on fetal NTDs in human subjects. The exposure under investigation was the factors associated with fetal NTDs. The comparison relied on measures such as odds ratio (OR) or related risk (RR) ratios reported in the included studies as non-risk factor. The primary outcome of interest was the exploration of the association between the identified factors and fetal NTDs.

The study selection itself involved two independent authors, E.J. and H.A., who meticulously screened the titles and abstracts of the identified studies, as well as the full texts, to determine their eligibility for inclusion. Any disagreements that emerged during this process were successfully addressed through negotiation and discussion.

### 2.4 Extraction of data

Two authors, E.J. and H.A., independently conducted the extraction of data from the main body of each article. In instances of discrepancies, conflicts were resolved by a third author, Z.M. The extracted data encompassed various elements, including the first author’s name, publication date, sample size, number of included studies in the meta-analysis, effect size, study design type, potential factors, confidence interval, and credibility of the evidence. Additionally, the *p*-value of the largest study, quality assessment, between-study heterogeneity, and bias were meticulously recorded.

### 2.5 Quality assessment analysis

We utilized the “Measurement Tool to Assess Systematic Reviews (AMSTAR2)” to assess the quality of the studies included in our analysis [15]. Disagreements among assessors were resolved using AMSTAR2, which consists of 16 questions (detailed in Table S3). Each item received a score of “yes,” “partial yes,” or “no.” Notably, items 2, 4, 7, 9, 11, 13, and 15 were designated as critical, carrying greater weight in the scoring process. The cumulative score derived from these critical questions determined the overall quality rating of the meta-analysis. This overall rating could be categorized as high, moderate, low, or critically low, providing a comprehensive assessment of the study’s quality and reliability.

### 2.6 Statistical analysis

We conducted all statistical analyses using R software, specifically version 4.0.5. In addition to the R base package, we employed various other R packages, including reporter, Metafor, ConfoundedMeta, xlsx, and epiR. To ensure the precision of our analyses, we reviewed the included meta-analyses based on information extracted from the sources, such as reported *p*-values, contingency tables, and sample sizes. In cases where necessary information was not available in the text, we contacted the corresponding authors of the original papers to request this data. If we did not receive a response, we excluded the original paper from the re-analysis process. For the computation of pooled effect sizes, we utilized a random-effects model during the re-analysis. Throughout our analyses, we set the significance level to 0.05. To assess the heterogeneity among studies, we employed both Cochrane’s *Q* test and the *I* ^2^ measure [16]. Substantial heterogeneity was considered when *I* ^2^ was 50% or higher. Additionally, we calculated the prediction interval, representing the potential range of estimated effect sizes in future studies. Furthermore, we conducted Egger’s regression test to examine the presence of a small study effect [17]. 

#### 2.6.1 Grading quality of evidence

The criteria used to stratify the evidence were as follows: Class I: convincing evidence, Class II: highly suggestive evidence, Class III: suggestive evidence, Class IV: weak evidence, and NS: not significant (Table 1).

**Table 1:** Criteria for level of evidence

Classification	Criteria
Convincing evidence (Class I)	1. More than 1,000 cases
	2. Significant summary associations (*p* < 10^−6^) per random-effects calculations
	3. No evidence of small-study effects (Egger < 0.1)
	4. No evidence of excess of significance bias
	5. Prediction intervals not including the null value
	6. Largest study nominally significant (*p* < 0.05)
	7. Not large heterogeneity (*I* ^2^ < 50%)
	8. Robust results based on sensitivity analysis
Highly suggestive evidence (Class II)	1. More than 1,000 cases
	2. Significant summary associations (*p* < 10^−6^) per random-effects calculation
	3. Largest nominally significant study (*p* < 0.05)
Suggestive evidence (Class III)	1. More than 1,000 cases
	2. Significant summary associations (*p* < 10^−3^) according to random effect calculations
Weak evidence (Class IV)	1. All other associations with *p* < 0.05
Non-significant associations (NS)	1. All associations with *p* < 0.05

### 2.7 Sensitivity analysis

Mathur’s method was employed to conduct a sensitivity analysis, evaluating the robustness of the meta-analysis [18]. This approach entails the computation of a bias factor and the assessment of the strength of paired confounding associations.


**Ethics approval:** The ethics committees of Hamadan University of Medical Sciences have approved this study with the reference number IR.UMSHA.REC.1402.634.

## 3 Results

**Figure 1:**
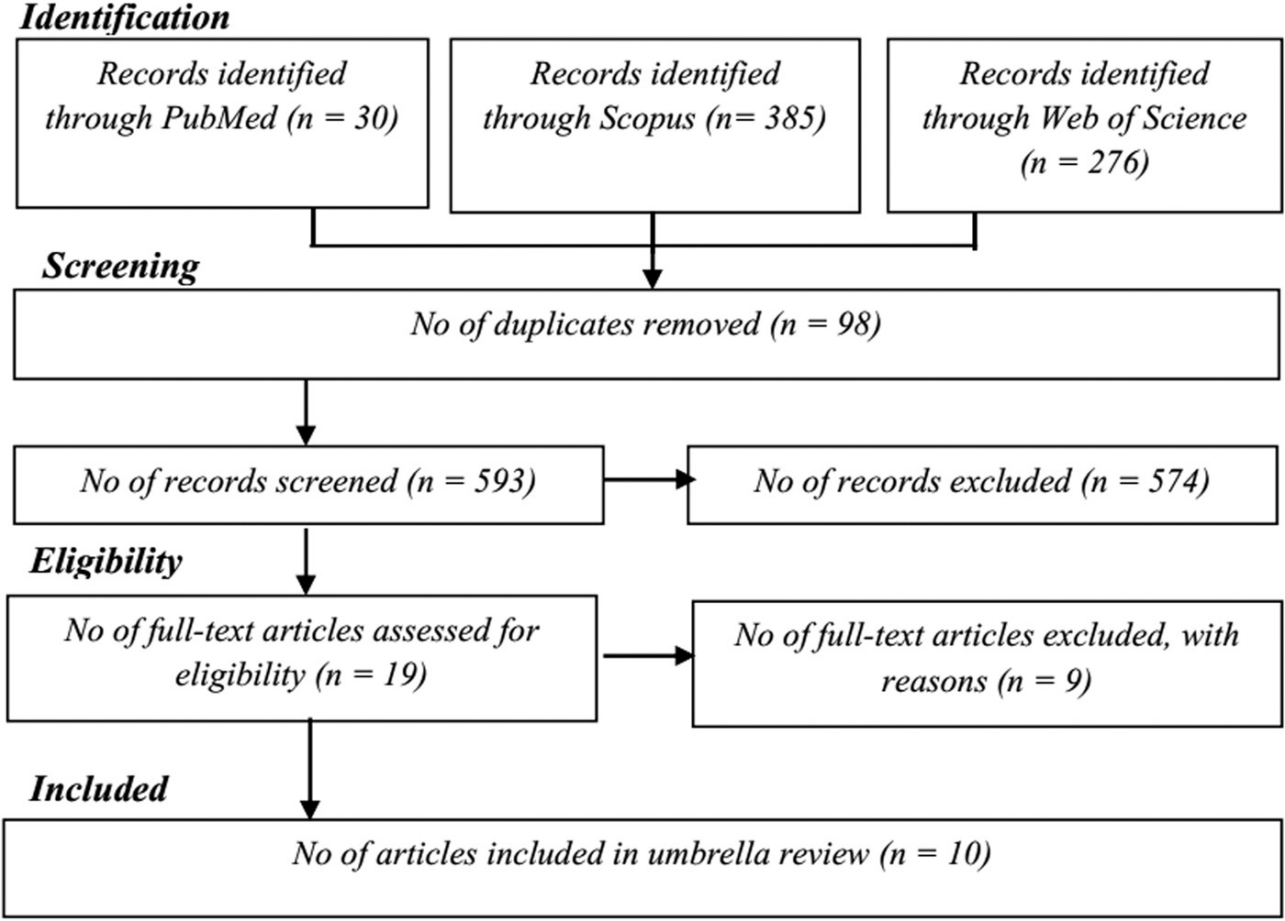
Process of the meta-analyses included in the umbrella review.

In the ten studies [5–8,19–22] encompassed in this analysis, a total of 12 meta-analyses were included, involving 40,612 cases of fetal NTDs with a sample size of 15,770,497 participants. The selection process for the meta-analyses included in this umbrella review is depicted in Figure 1, and the reasons for excluding certain meta-analyses are detailed in Table S2.

The studies integrated into this umbrella review comprised both cohort and case–control designs, constituting 137 original studies, including 25 cohort studies and 112 case–control studies. Within the scope of this umbrella review, we identified 12 factors: alcohol consumption during pregnancy, maternal overweight, maternal underweight, coffee consumption during pregnancy, hyperthermia, influenza, multivitamin supplementation, maternal dietary nitrate intake, obesity, smoking, passive smoking, and low maternal vitamin B12 (Table 2). Among these 12 associations examined, six exhibited statistically significant *p*-values when analyzed using the random-effects model. Notably, ten studies included in this analysis involved at least 1,000 cases of fetal NTDs. Additionally, three of these studies reported heterogeneity (*I* ^2^) of less than 50%, one study showed small study effects, and five studies displayed an excess significance bias (Table 3).

**Table 2:** Maternal factors for NTDs in the umbrella review

Risk factors	Source (year)	Number of population	Number of included studies	Study design	Effect metrics	Random effect summary estimate	AMSTAR1 quality	Credibility of evidence
Alcohol consumption during pregnancy	Leng (2016)	21,489	8	Case–control/cohort	OR	1.01 (0.71, 1.45)	Critically low	Not significant
Maternal overweight	Vena (2022)	1,106,644	9	Case–control/cohort	OR	1.09 (0.92, 1.30)	Critically low	Not significant
Maternal underweight	Vena (2022)	809,592	8	Case–control/cohort	OR	1.34 (0.73, 2.47)	Critically low	Not significant
Coffee consumption during pregnancy	Li (2016)	12,344	7	Case–control/cohort	OR	0.86 (0.51, 1.45)	Critically low	Not significant
Hyperthermia	Moretti (2005)	39,617	15	Case–control/cohort	OR	1.92 (1.61, 2.29)	Low	Highly suggestive
Influenza	Luteijn (2014)	28,976	11	Case–control/cohort	OR	3.33 (2.05, 5.40)	Low	Suggestive
Multivitamin supplementation	Goh (2006)	10,254	8	Case–control	OR	0.76 (0.60, 0.96)	Critically low	Weak
Maternal dietary nitrate intake	Rahimi Kakavandi (2018)	3,049	5	Case–control	RR	1.33 (0.89, 1.99)	Critically low	Not significant
Obesity	Huang (2017)	1,288,077	24	Case–control	OR	1.68 (1.51, 9.44)	Critically low	Highly suggestive
Smoking	Meng (2018)	12,393,207	21	Case–control/cohort	OR	1.05 (0.90, 1.22)	Critically low	Not significant
Passive smoking	Meng (2018)	55,097	12	Case–control/cohort	OR	1.90 (1.58, 2.31)	Critically low	Highly suggestive
Low maternal vitamin B12	Wang (2012)	2,151	9	Case–control	OR	2.41 (1.90, 3.06)	Critically low	Weak

Class II: highly suggestive; Class III: suggestive; class IV: weak; NS: not significant.

**Table 3:** Assessment of the evidence credibility for maternal factors associated with NTDs

Risk factors	Number of cases	Summary associations (*p*-value) per random-effects calculations	Small-study effects (*p*-value for Egger)	Excess of significance bias (*p*-value)	Prediction intervals	Largest study nominally significant (*p*-value)	Heterogeneity (*I* ^2^%)	Sensitivity analysis	Classification
Alcohol consumption during pregnancy	2,984	0.574	0.707	0.037	0.39, 3.27	<0.001	53.5	*T* = 1.038, *G* = 1.236	Not significant
Maternal overweight	3,685	0.304	0.583	0.696	0.72, 1.65	<0.001	24.6	*T* = 0.979, *G* = NAN	Not significant
Maternal underweight	2,098	0.342	0.855	0.286	0.25, 7.32	<0.001	74.1	*T* = 1.235, *G* = 1.774	Not significant
Coffee consumption during pregnancy	1,159	0.830	0.680	0.439	0.20, 4.38	0.047	86.4	*T* = 1.265, *G* = 1.844	Not significant
Hyperthermia	10,344	<0.000001	0.023	0.725	1.58, 2.33	<0.000001	0.0	*T* = 2.668, *G* = 4.779	Highly suggestive
Influenza	2,229	0.0001	0.333	0.004	0.61, 15.85	0.002	61.17	*T* = 2.401, *G* = 4.237	Suggestive
Multivitamin supplementation	1,829	0.003	0.347	0.996	0.42, 1.35	0.002	69.4	*T* = 1.467, *G* = 2.294	Weak
Maternal dietary nitrate intake	910	0.416	0.108	0.600	0.38, 4.66	0.059	80.0	*T* = 1.241, *G* = 1.788	Not significant
Obesity	5,847	<0.000001	0.944	0.003	1.23, 2.30	<0.000001	37.8	*T* = 1.567, *G* = 2.511	Highly suggestive
Smoking	5,683	0.749	0.892	0.536	0.55, 1.9	0.947	57.6	*T* = *T* = 1.000, *G* = 1.020	Not significant
Passive smoking	3,259	<0.000001	0.117	0.047	1.51, 1.9	<0.000001	50.5	*T* = 1.197, *G* = 1.682	Highly suggestive
Low maternal vitamin B12	585	<0.000001	0.094	0.039	1.02, 9.06	0.006	47.0	*T* = 3.257, *G* = 5.969	Weak

Class II: highly suggestive; Class III: suggestive; class IV: weak; NS: not significant.

The sensitivity analyses unveiled that three of the meta-analyses (multivitamin supplementation, obesity, and passive smoking) were relatively sensitive to unmeasured confounding, as evidenced by a bias factor of less than 1.75 in each of their included studies. These meta-analyses had the potential to decrease the proportion of studies with a true OR exceeding 1.1 to less than 20%. On the contrary, the remaining three meta-analyses (low maternal vitamin B12, influenza, and hyperthermia) demonstrated relative robustness to unmeasured confounding, with a bias factor exceeding 1.90 in each of the included studies. This level of robustness could reduce the percentage of studies with a true OR greater than 1.1 to less than 10%.

Notably, alcohol consumption during pregnancy, maternal overweight, maternal underweight, coffee consumption during pregnancy, maternal dietary nitrate intake, and smoking did not emerge as significant risk factors in this context (Table 3).

Three risk factors for fetal NTDs, namely hyperthermia with an OR of 1.92 (95% CI: 1.61, 2.29), obesity with an OR of 1.68 (95% CI: 1.51, 9.44), and passive smoking with an OR of 1.90 (95% CI: 1.58, 2.31), were classified as highly suggestive evidence (Class II). Influenza, with an OR of 3.33 (95% CI: 2.05, 5.40), was considered a risk factor with suggestive evidence (Class III). Multivitamin supplementation during pregnancy, with an OR of 0.76 (95% CI: 0.60, 0.96), and low maternal vitamin B12, with an OR of 2.41 (95% CI: 1.90, 3.06), were categorized as weak evidence (Class IV).

Alcohol consumption during pregnancy, with an OR of 1.01 (95% CI: 0.71, 1.45), maternal overweight, with an OR of 1.09 (95% CI: 0.92, 1.30), maternal underweight, with an OR of 1.34 (95% CI: 0.73, 2.47), coffee consumption during pregnancy, with an OR of 0.86 (95% CI: 0.51, 1.45), maternal dietary nitrate intake, with a RR of 1.33 (95% CI: 0.89, 1.99), and smoking, with an OR of 1.05 (95% CI: 0.90, 1.22), were not identified as risk factors for fetal NTDs.

Crucially, the quality of the included meta-analyses was critically low (ten meta-analyses) and low (two meta-analyses) based on AMSTAR2 (Tables 3 and S3).

## 4 Discussion

### 4.1 Interpretation of the results

In this umbrella review, we summarized the available evidence from a total of 12 meta-analyses with 40,612 cases of fetal NTDs with 15,770,497 participants. Based on the results of our research, hyperthermia, obesity, passive smoking, influenza, and low maternal vitamin B12 were identified as risk factors for NTDs. Multivitamin supplement during pregnancy was a protective factor for fetal NTDs. Hyperthermia, obesity, and passive smoking were classified in the category of highly suggestive evidence (Class II). In these three association, only one criterion was not met, small-study effects for hyperthermia and excess of significance bias for obesity and smoking were significant (statistical significance; *p* < 0.05). In addition, influenza was considered a risk factor with suggestive evidence (Class III), and no meta-analysis was classified as convincing evidence (Class I).

### 4.2 Comparison to the literature

Heterogeneity between studies was higher than 50% for alcohol consumption during pregnancy, maternal underweight, coffee consumption during pregnancy, influenza, multivitamin supplementation, maternal dietary nitrate intake, smoking, and passive smoking. This difference can also be caused by the fact that there are two or more groups of studies in our data that have a different true effect and it can lead to reducing the generalizability of the estimated overall effect to all of the included studies [23].

The hypothesis that maternal hyperthermia is teratogenic during pregnancy was first investigated in a series of animal studies. Previous evaluations regarding maternal fever, have confirmed that the high core body temperature in pregnant mothers (especially in the first trimester), mainly affects the face and brain and is also associated with an increased risk for congenital malformations and NTDs [24,25]. High core body temperatures in humans can be due to fever caused by viral or bacterial diseases (e.g., malaria, rubella, varicella, and cytomegalovirus), and also high fever may lead to adverse pregnancy outcomes through poor maternal nutrition. The teratogenicity of hyperthermia can be partially justified through infectious agents and insufficient nutrition [26]. Also in our study, influenza was identified as the strongest teratogenic factor on NTD. Influenza can lead to viral infection of the fetus or it may have negative effects through hyperthermia, fever, or the use of antiviral and antipyretic drugs in some mothers [27].

Previous studies that have examined the association between obesity and NTDs have sometimes provided conflicting results, this could be due to small sample sizes, the use of different cut-off points to define obesity, or different body characteristics of women [28,29]. Obesity can lead to NTDs through different mechanisms, for example, increased rates of hypertensive diseases (chronic hypertension and preeclampsia), pre-gestational and gestational diabetes, poorer nutrition including folic acid deficiency in diet, cesarean section, and infections may negatively affect pregnancy outcome [30]. Metabolic disorder may actually play a role. Obesity is usually characterized by insulin resistance and latent or overt hyperglycemia, possibly related to birth defects, as occurs in diabetes. These problems are less common in overweight women. Therefore, the association between overweight and the risk of NTDs was not significant.

Passive smoking or secondhand cigarette smoke is the involuntary inhalation of environmental tobacco smoke by a non-smoker [31]. The results of previous animal and human studies find that the components of cigarette smoke, including nicotine, can pass through the placenta and disrupt the stability of the placental barrier by causing problems in the expression of the tight junction proteins [32].

In recent years and in several studies, the protective effect of folic acid-fortified multivitamins on congenital anomalies, including NTDs and risk of orofacial clefts, has been suggested [33]. Several factors are essential for the mechanism of folate effect on NTDs, one of these modifiable factors is vitamin B12. Knowledge of this biochemistry and some similarities in the clinical features of folate and vitamin B12 deficiency can confirm the hypothesis that these two vitamins are risk factors for NTDs [34].

The results of the sensitivity analysis indicate that the observed associations between multivitamin supplementation, passive smoking, obesity, and NTDs are affected by unadjusted confounders and lead to bias, this indicates that the ORs reported in some studies may be not real. In fact, in the individual studies included in these meta-analyses, only some confounders were controlled, and other unknown confounders may have affected the results.

Alcohol consumption during pregnancy was not considered a risk factor for NTDs. This could be due to consideration of the various terms of NTDs types, control population, and exposure categorization. After performing a sensitivity analysis in the meta-analysis, the result was largely unchanged. In addition, this meta-analysis study further determined the effects of alcohol on the spina bifida subtype of NTDs.

### 4.3 Strengths and limitations

An umbrella review provides an overall picture of consistent or conflicting findings around a topic and is useful to inform guidelines and clinical decision-making [35]. This study was the first umbrella review to examine risk factors for NTDs. However, the limitations that should be considered in the interpretation of its results are as follows: (1) the quality of all studies was low or critically low based on the AMSTAR2 quality assessment tool and the quality of any umbrella review depends on its included meta-analyses. The low quality of studies can indicate a degree of selection bias, information bias, or lack of control and adjustment of potential confounders in individual studies that investigated NTDs risk factors. (2) The search strategy was limited to three databases including PubMed, Web of Science, and Scopus and we did not have access to other databases.

## 5 Conclusion

In this umbrella review, we identified four risk factors including hyperthermia, influenza, obesity, and passive smoking as suggestive or highly suggestive evidence for NTDs. Low maternal vitamin B12 was identified as a risk factor for NTDs, supported by weak evidence. Additionally, there is weak evidence suggesting that multivitamin supplementation acts as a protective factor against NTDs.

## Supplementary Material

Supplementary Table
