# Supplementary material for: Maternal factors for neural tube defects in offspring: An umbrella review
Source: Open Med (Wars). 2024 Oct 14;19(1):20241061. doi: 10.1515/med-2024-1061 (PMC11491881; doi:10.1515/med-2024-1061)
Supplement: Supplementary Table [file med-2024-1061-sm.pdf]

Supplementary material

Table S1: Search strategy

1. Influencing factors or related factors or risk factors
1. *neural tube defects OR* spina bifida *OR* anencephaly *OR Encephalocele OR* NTDs
1. Systematic review or Meta-analysis or Meta analysis or synthesis
1. #1 and #2 and #3

Table S2: Excluded references with reasons

***Outcome already explored in larger meta-analysis* (*n* = 7)**

1. Donnan J, Walsh S, Sikora L, Morrissey A, Collins K, MacDonald D. A systematic review of the risks factors associated with the onset and natural progression of spina bifida. Neurotoxicology. 2017;61:20-31.
2. Hackshaw A, Rodeck C, Boniface S. Maternal smoking in pregnancy and birth defects: a systematic review based on 173 687 malformed cases and 11.7 million controls. Human Reproduction Update. 2011;17(5):589-604.
3. Jia S, Wei X, Ma L, Wang Y, Gu H, Liu D, et al. Maternal, paternal, and neonatal risk factors for neural tube defects: A systematic review and meta-analysis. International journal of developmental neuroscience : the official journal of the International Society for Developmental Neuroscience. 2019;78:227-35.
4. Nicoletti D, Appel LD, Neto PS, Guimaraes GW, Zhang LJ. Maternal smoking during pregnancy and birth defects in children: a systematic review with meta-analysis. Cadernos De Saude Publica. 2014;30(12):2491-529.
5. Stothard KJ, Tennant PWG, Bell R, Rankin J. Maternal overweight and obesity and the risk of congenital anomalies: A systematic review and meta-analysis. Jama. 2009;301(6):636-50.
6. Wang M, Wang ZP, Gong R, Zhao ZT. Maternal smoking during pregnancy and neural tube defects in offspring: A meta-analysis. Child's Nervous System. 2014;30(1):83-9.
7. Wang M, Wang ZP, Zhang M, Zhao ZT. Maternal passive smoking during pregnancy and neural tube defects in offspring: A meta-analysis. Archives of Gynecology and Obstetrics. 2014;289(3):513-21.

***NTDs did not estimated with OR/RR* (*n* = 1)**

1. Yang M, Li W, Wan ZH, Du YK. Elevated homocysteine levels in mothers with neural tube defects: a systematic review and meta-analysis. Journal of Maternal-Fetal & Neonatal Medicine. 2017;30(17):2051-7.

***Full paper did not find* (*n* = 1)**

1. AR Vieira, SC Taucher. Influence of maternal age on the risk for neural tube defects, a meta analysis, Revista Medica de Chile. 2005.

Table S3: Quality of studies based on AMSTAR2 items

Rating	16	15	14	13	12	11	10	9	8	7	6	5	4	3	2	1	Items study
Critically low	Y	PY	PY	PY	N	Y	N	Y	Y	N	Y	Y	Y	Y	N	Y	Leng (2016)
Critically low	N	N	PY	N	N	Y	N	Y	Y	N	Y	Y	Y	Y	Y	Y	Vena (2022)
Critically low	Y	N	N	N	N	Y	N	Y	PY	N	Y	Y	PY	Y	N	Y	Li (2016)
Low	N	PY	N	PY	N	Y	N	Y	Y	Y	Y	Y	PY	Y	N	Y	Moretti (2005)
Low	N	Y	PY	Y	N	Y	N	Y	Y	Y	Y	Y	Y	Y	N	Y	Luteijn (2014)
Critically low	Y	N	PY	N	N	Y	N	PY	PY	N	Y	N	PY	Y	N	Y	Goh (2006)
Critically low	Y	N	N	N	Y	Y	N	Y	Y	N	Y	Y	Y	Y	N	Y	Rahimi Kakavandi (2018)
Critically low	N	PY	N	N	N	Y	N	PY	PY	N	Y	Y	Y	Y	N	Y	Huang (2017)
Critically low	N	N	PY	PY	PY	Y	N	Y	Y	N	Y	Y	Y	Y	N	Y	Meng (2018)
Critically low	Y	Y	N	N	N	Y	N	PY	Y	N	N	N	Y	Y	N	Y	Wang (2012)

Y: Yes; pY: Partial yes; N: No.

AMSTAR 2 items evaluated: 1 PICO consideration in the research question and inclusion criteria. 2 Protocol establishment beforehand. Any deviations? 3 Explaining if/why only certain study designs were included. 4 Comprehensive search. 5 Two persons performed the search.

6 Two persons extracted the data. 7 Providing the list of exclusion with reasons.

8 Presenting all details of the included papers. 9 Proper technic for assessing the risk of bias. 10 Reporting sources of funding. 11 Appropriate statistical methods.

12 Assessment of the potential impact of risk of bias in individual studies on the results of the meta-analysis. 13 Assessment of the potential impact of risk of bias in individual studies on the discussion of the meta-analysis. 14 Discussion of heterogeneity of the results of meta-analysis. 15 Investigation of publication bias. Have they influenced the results? 16 Reports of potential conflict of interest.

Items 2, 4, 7, 9, 11, 13, and 15 are the critical items. For High quality: 0–1 non-critical weakness. Moderate quality: >1 non-critical weakness. Low quality: 1 critical flaw with or without non-critical weaknesses. Critically low quality: >1 critical flaw with or without non-critical weaknesses.
